# Nanoparticle Clearance and New Horizons in Engineered Drug Delivery

**DOI:** 10.3390/pharmaceutics18040471

**Published:** 2026-04-13

**Authors:** Bryan J. Mathis, Alexander Zaboronok, Ying Shi, Yoshiyuki Nagumo, Hiroyuki Nishiyama, Yuji Hiramatsu

**Affiliations:** 1 Department of Cardiovascular Surgery, Institute of Medicine, University of Tsukuba, Tsukuba 305-8575, Japan; 2 Department of Neurosurgery, Institute of Medicine, University of Tsukuba, Tsukuba 305-8575, Japan; a.zaboronok@md.tsukuba.ac.jp; 3 Department of Urology, Union Hospital, Tongji Medical College, Huazhong University of Science and Technology, Wuhan 430074, China; 4 Department of Urology, Institute of Medicine, University of Tsukuba, Tsukuba 305-8575, Japan

**Keywords:** nanoparticles, drug clearance, polymer, nanomedicine, metabolism

## Abstract

Nanomedicine has advanced rapidly as engineered nanoparticles have become increasingly capable of improving drug stability, targeting, controlled release, and biocompatibility. However, nanoparticle clinical utility relies on both delivery efficiency and how they are metabolized, retained, and cleared. This review examines the major biological pathways governing nanoparticle clearance and discusses how engineering parameters can be tuned to influence bioaccumulation, metabolism, excretion, and therapeutic performance with a wide range of available materials. This article is a narrative review of the recent and foundational literature on medically relevant nanoparticles, including lipid-based, polymeric, biopolymer, inorganic, polylactide, and bile-derived systems. All relevant translational, biochemical, chemical, and clinical literature from PubMed was searched from January 1971 to January 2026 to obtain a representative sample of work before information extraction. Nanoparticle clearance is governed by interconnected molecular and organ-level processes that vary according to composition, size, surface chemistry, and route of administration. Surface modifications with PEGylation, zwitterionic coatings, cholesterol, proteins, or responsive linkers can prolong circulation, alter immune recognition, and direct organ-specific handling. While rapid clearance remains desirable for many systemically acting drugs, prolonged intracellular or intratumoral retention may improve outcomes, particularly in boron neutron capture therapy and other activation-dependent treatments. Nanoparticle clearance should be regarded as a context-dependent design parameter rather than a universal limitation. Rational control of clearance kinetics may improve both safety and therapeutic effectiveness in next-generation engineered drug delivery systems.

## 1. Introduction

### 1.1. Nanomedicine and Considerations

Nanomedicine has made evolutionary progress over the last 20 years, culminating in the recent release of the Comirnaty COVID-19 mRNA vaccine, which contained engineered lipid nanodroplets. Before mRNA vaccines, a landmark in the development of nanomedicines was the introduction of liposomal doxyrubicin for cancer treatment, a drug that has been used extensively in metastatic breast and other reproductive cancers [1]. However, its use in other cancers has yielded ambiguous results and has not been tested extensively in somatic cancers. Thus, the next stage was the use of interfering RNA to reduce levels of disease protein targets. Onpattro^®^ (patisiran) (RNA interference therapy) for hereditary transthyretin-mediated (hATTR) amyloidosis was shown to successfully suppress neuropathy symptoms in the phase 2 and 3b OLE and APOLLO studies, highlighting the utility of targeted nanomedicine and driving interest in a new class of mRNA nanomedicines for vaccine purposes [2,3]. Centered on the concept of particles sized 1–1000 nm, nanomedicine has become an attractive research field to overcome traditional obstacles involved in pharmaceutical manufacturing (such as maintaining a stable salt form, capable of being metabolized by the liver). Clinically, nanomedicines are attractive for overcoming typical issues encountered in daily practice, including toxicity, off-target exposure, poor absorption, lack of controlled release, inability to be co-delivered with combined therapy, and undesired immunogenic effects. Now that diverse raw materials can be engineered for a panoply of specifications, including degradation dynamics, cargo type, biological compatibility, and secondary effects, much effort is currently expended on developing highly holistically compatible nanomedicines. This is evident in the new drug pipeline, with at least 77 Phase II and 68 to 80 Phase III nanomedicines currently being tested [4]. The emphasis on development of engineered solutions signals a paradigm shift in drug design where classical pharmacokinetics no longer need apply; poorly soluble drugs can be encapsulated in amphipathic coatings, layered nanoparticles may resist degradation and deliver cargo to specific areas or over defined time periods, bioaccumulation can increase dose-dependent effects or accumulation before purposeful activation, co-delivery with combined therapy within the same particle is feasible, and immune evasion to prevent inflammation can be controlled via surface ligands [5,6,7]. Doxil was an excellent early example of enhancing therapy with nanoengineering; the use of polyethylene glycol-liposomal nanoparticles allowed for increases of up to 90-fold in bioavailable doxorubicin, even 7 days after administration [8].

**Figure 1:**
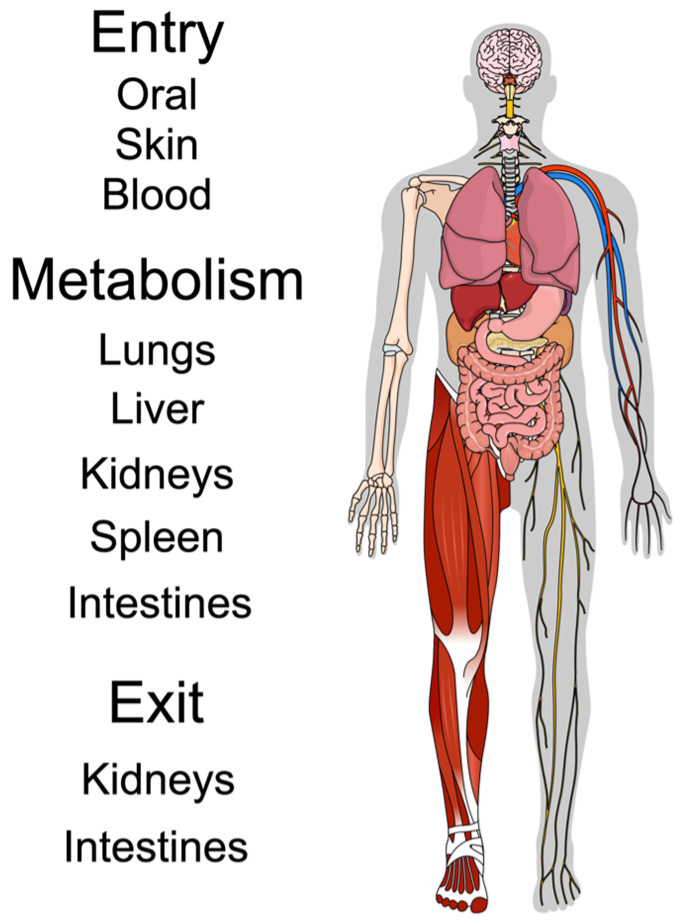
Primary routes of entry, metabolism, and exit for nanoparticles.

Generally, nanoparticles are first designed around their intended cargo and are commonly made from bio- and holistically compatible organic polymers, such as poly(lactic-co-glycolic acid) (PLGA) [9]. Recently, oxide nanoparticles (made from silica, iron, or ceramics) have been gaining traction for diverse uses: magnetic oxides may serve as less-toxic contrast agents in medical imaging, while ceria oxides with catalytic ability may neutralize reactive oxygen species, and silica oxides with defined pore sizes can exploit diffusion to release cargo at precise time points [10]. There is also an array of modified lipids that exploit the properties of cholesterol, phospholipids, or polymerized glycols to deliver nucleic acid cargo directly into cells, bypassing the membrane [11]. All nanoparticles for medical purposes are designed around the same criteria, namely, biocompatibility, targeting, cargo delivery, and functionality. This review details the general aspects of nanoparticle metabolism from a clinically relevant standpoint while providing a broad survey of attributes and modifications that can change the functional capacity and systemic-level (holistic) compatibility of nanoparticles intended for use in medicine (Figure 1).

### 1.2. From Biocompatibility to Holistic Compatibility

Biocompatibility is generally defined at the cellular level and encompasses a lack of cytotoxicity, immune reactivity, and oxidative stress. However, here, we propose a compatibility that goes beyond the cellular effects to the holistic (whole-body) level, which encompasses non-toxicity, immune interface capability (or lack thereof), and excretion. This “holistic compatibility” concept becomes an important safety parameter for ensuring that nanoparticles will accumulate in therapeutically relevant amounts, evade the host immune system to prevent inflammation or subsequent adverse reactions through sensitization, and be cleared from the system through natural excretion routes. It also ensures that bioaccumulation to therapeutic levels is accomplished while avoiding toxicity. Nanoparticles that avoid interactions with off-target proteins and immune cells/factors are often termed “furtive”, “cloaked”, or “stealthy”, and this capability is highly desirable for clinical use.

### 1.3. Targeting

Targeting ensures the accurate delivery of the cargo to the intended location and can be active, through ligand binding to cell-surface receptors, or passive, taken up through gradients or membranes and past epithelial barriers. The blood–brain barrier (BBB) is a good example of such an epithelial obstacle, and recently, a 72-lipid mRNA nanocarrier has been demonstrated to cross the barrier and bioaccumulate in the brain [12].

### 1.4. Cargo Delivery

Nanocarriers must be small enough to diffuse through vascular networks but large enough to carry useful cargo of sufficient size and capacity. Having a high surface-to-volume ratio allows for larger capacity, while release factors may depend on physicochemical properties such as pH changes, heat, enzymatic digestion, or interaction with hydrophobic/philic microenvironments. An additional concern is that drugs with poor hydrophobicity must be stabilized to ensure steady and reliable delivery.

### 1.5. Functionality

Once at the correct target, nanoparticles can be engineered to release their payload as a primary function but may also have multiple secondary functions. For example, a layered oxide nanoparticle may be injected to provide contrast for imaging while releasing a drug through a polymeric coating and targeting cancer cells with a functional ligand. More complex systems may eventually combine therapies (e.g., antiretroviral therapy) within a single nanoparticle to ensure stoichiometric parity in release and effect. Table 1 gives representative studies featuring varied types of nanoparticles, their applications, and the modifications required to make them functional.

## 2. Metabolism of Nanoparticles

**Figure 2:**
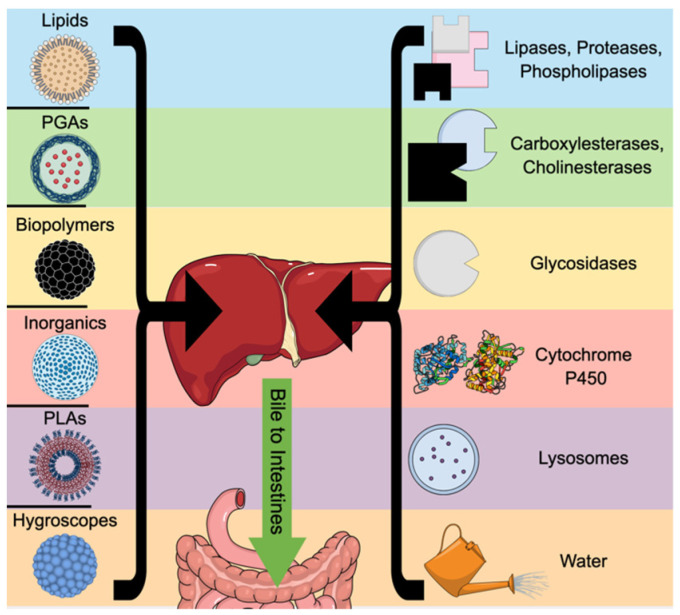
Molecular clearance: The liver contains diverse enzymes and an aqueous environment to metabolize nearly any nanoparticle at the molecular level, breaking down particles over time to incorporate them into bile for excretion. PGAs include polyglutamic and polyglycolic acids; biopolymers include chitosan, dextran, and hyaluronic acids; inorganics include silica, gold, and carbon; PLAs include all polylactic acids; and hygroscopes include water-attracting compounds such as bile salts and wetting agents such as Tween.

Metabolism occurs in two primary dimensions, macro and molecular, with macro being systemic (or at least organ-level) and molecular occurring within target cells. The primary organs involved in excretion of nanomedicines are the liver, spleen, kidneys, and intestines, each of which may contain one or more molecular processes. Clinically, since an inability to be metabolized may result in unwanted bioaccumulation/biotoxicity or drug–drug interactions due to persistence in organs, metabolic considerations are at the forefront of nanomedicine design. Figure 2 displays an example of how nanoparticles are metabolized, degraded, and cleared within the liver by these molecular-level forces.

### 2.1. Clearance Step 1: Molecular Level

#### 2.1.1. Enzymes

Nanomedicines may be exposed to enzymatic degradation/metabolism throughout the digestive tract, especially if orally administered, but liver enzymes are also an important part of nanoparticle processing. Carboxylesterases and cholinesterases within the liver and intestines can digest polymerized glycolic acids and lipids, while lipases, proteases, and phospholipases in the liver cleave liposomes, engineered lipids, and nanoparticles with albumin or peptide layers [46,47]. Glycosidases target chitosan, dextran, or glycols/hyaluronic acid, and oxidation by liver-resident oxidases (e.g., cytochrome P450 or NADPH oxidase) can attack inorganic particles [48]. Several layers of starch or resistant coatings may be exploited to slow digestion by enzymes. However, inorganic nanoparticles may interfere with cytochrome enzymes even as they are oxidized by them, making the use of carbon, silica, zinc/titanium dioxide, and gold/silver particles in treatments a point of concern [49].

#### 2.1.2. pH

Acidic environments, such as the inside of the stomach, may rapidly degrade polymerized nanoparticles, but pH-responsive elements may be useful for drug delivery. The pH variation from relatively neutral blood (~pH 7.4) to the acidic interior of active lysosomes (pH of 5.0 or lower) can be exploited by using charge-shifting polymers, like poly(2-diisopropylamino ethyl methacrylate) (PDPA), that swell and release cargo once taken up by cells into lysosomes [50]. Polylactides and polyurethane ureas may also be readily degraded and release acidic intermediates that further lower pH in the microenvironment [51]. As the general body environment tightly regulates pH outside of specified organs (such as the stomach), pH-stable coatings may increase resistance to passive digestion during slightly alkaline or acidic fluctuations while en route to targeted treatment areas.

#### 2.1.3. Solubility Resistance in Payload and Particle

Low drug solubility is a frequent issue in standard pharmaceutical development, as most formulations are hydrophobic, and around 40% of current medicines are poorly soluble, which greatly affects their bioavailability [52]. Emulsions made with bile salts or wetting agents such as Tween may increase solubility and hydrophilic layers within liposomes, or polyethylene glycol can rectify poor bioavailability from low-soluble particles [52].

Solubility may also directly affect the rate of renal clearance, as the glomerular filtration barrier excludes particles larger than around 6 nm, and the secondary glomerular basement membrane excludes negatively charged particles [5]. However, these barriers can be bypassed, even by larger and less soluble spent nanoparticles if they are fragmented or by nanoparticles with non-spherical shapes. It has been reported that insoluble, long nanorods usually have a very small diameter (1–2 nm) and can pass through epithelial barriers [5]. Additionally, glycans found in virions may aid in bypassing this barrier and work synergistically with biodegradable polymers (e.g., PLGA, PLA) to flush spent nanoparticles through the kidneys [5]. Thus, although solubility is an important criterion for renal clearance, engineered systems may bypass it while remaining hydrophobic. Such nanoparticles are of use in stabilizing hydrophobic compounds for delivery.

For the nanoparticles themselves, solubility is a critical aspect of persistence (biodurability) since dissolved particles generally change quickly into ionic or molecular species that may change the local pH or other microenvironmental parameters, while slowly dissolving particles have greater retention and bioaccumulation [53,54,55]. Biodurability/biopersistence of a selection of inorganic nanoparticles was shown in a report by Di Cristo and colleagues to be highest in titanium dioxide, followed by silicon dioxide and zinc oxide; this supports the concept of persistence being closely related to nanoparticle composition [56]. However, solubility is a multi-aspect factor since it is affected not only by composition but by particle size, surface area, shape, and the intended use case, in which biological parameters (including pH, ionic strength, and temperature) may play a role in solubilization [54]. Within inorganic nanosystem design, these solubility factors are highlighted, since silica-based nanocarriers can be constructed with increased internal porosity that shifts a relatively insoluble oxide toward a more biodegradable construct (Stöber mesoporous silica nanoparticles); these hollow constructs degrade from the interior outward [57]. Meanwhile, iron-doped silica strategies and silica–calcium phosphate hybrid designs are being engineered to dissolve under specific biological or acidic conditions [58,59]. In nanosystem engineering, solubility is also related to size: downstream clearance is highest in particles less than 5.5 nm in size that pass through the glomerular filters in the kidneys, while even a 0.4 nm increase in zwitterionic gold nanoparticle diameter due to additional surface glycine (but without compensatory surface modification) significantly reduced clearance and increased persistence [60,61].

Surface chemistry greatly affects bioaccumulation through modification of hydrophobicity and, thus, solubility. Iron oxide nanoparticles coated with a PEG biopolymer were shown to accumulate mainly in the murine liver and spleen, with slow biodegradation/clearance over two weeks and no obvious toxicity, whereas polyethylenimine-coated analogs had both higher uptake and dose-dependent lethal toxicity through reactive oxygen species generation [62]. Similarly, biodegradable organic carriers also rely on polymer composition to govern hydrolytic degradation through regulation of solubility in vivo; the ester linkages of PLGA are readily hydrolyzed in water, and the resultant dissolution can affect persistence, but the chemical addition of methyl groups can be exploited to adjust hydrophobicity [63].

#### 2.1.4. Immunogenicity

The immune system mainly responds to foreign molecules, but it also acts as a de facto second digestive system in that it may also clear damaged tissue through phagocytosis and stimulate healing in a classic M2 response. Professional antigen-presenting cells (APCs; e.g., macrophages, dendritic cells) engulf particles and digest them in acidic lysosomes. Such processed foreign proteins are presented via major histocompatibility complex (MHC) protein II clusters on the APC cell surface to educate and stimulate expansion of effector cells (T cells). Somatic cells may also take up lipid-based nanoparticles and present proteins on their cell surfaces to stimulate the immune response and generate antibodies. Thus, nanoparticles may exploit this “micrometabolic” effect, as seen with the recent COVID-19 vaccine, in which mRNA is delivered to myocytes via lipid nanoparticles, and the resultant generated spike protein is presented on the cell surface to stimulate an immune response [64]. Research is also being conducted on immunologically neutral nanoparticles; these furtive systems incorporate phosphatidylserine, antigenic peptide sequences (e.g., MHC proteins), cell-surface molecules that will generate immune tolerance, or immunosuppressive drugs (such as methotrexate or steroids) [65]. These furtive particles can thus (1) evade host metabolism long enough to either deliver a payload or (2) induce tolerance to a protein to reduce reactivity (i.e., anti-self-antigens in multiple sclerosis).

#### 2.1.5. Inert

Inert nanoparticles are usually inorganic and are derived from elements or compounds such as titanium dioxide, zinc oxide, gold, silver, silica, carbon, etc. While some oxidation may occur through NADPH oxidases or cytochrome P450 enzymes, these particles may not be completely destroyed by the body’s metabolism (or are resident for extended periods) and often end up being filtered by the spleen and kidneys, with the collection point being the former if the inert particles are immunoreactive (i.e., taken up by APCs and then shuttled to the spleen) and the latter if resident in the blood and below a certain cutoff size (approx. 5.5 nm) [66]. Inorganic nanoparticles are effectively recognized by macrophages, and extended lysosomal activity and oxidation by immune superoxides may dissolve even gold nanoparticles over time [67].

### 2.2. Clearance Step 2: Macro Level

#### 2.2.1. Liver/Intestines

**Figure 3:**
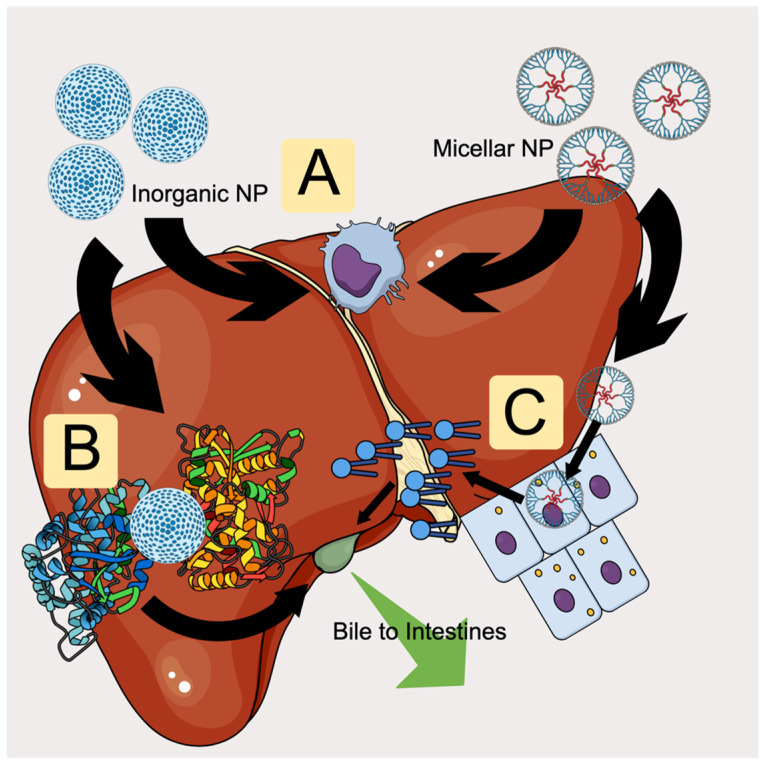
Degradation pathways of hypothetical nanoparticles in the liver. A: Inorganic and micellar nanoparticles may be taken up by patrolling antigen-presenting cells, such as macrophages (pictured). B: Inorganic nanoparticles can be oxidized by cytochrome P450 pathway enzymes before draining into the bile duct. C: Micellar or lipid-containing nanoparticles are digested by hepatocytes, and cellular waste is drained into the bile. NP = nanoparticle.

The liver is the primary metabolic organ in the body and can excrete waste products by solubilizing them in bile that drains into the intestines, in addition to generating urea from ammonia that passes from the body through urine. As all circulating blood passes through the liver and all consumed nutrition enters it through the hepatic portal vein, any nanoparticles that enter through oral or blood routes will eventually encounter the liver. Of particular importance to development of nanomedicines are lipid nanoparticles, which bind to apolipoprotein E (ApoE) and are targeted to hepatocytes by lipoprotein receptors, and the Kupffer cells (liver-resident macrophages) that take up nanoparticles as they pass through [68]. However, such sequestration by immune cells may allow particles to persist by evading the hepatobiliary excretion system. Bile, the primary waste product of the liver and an important emulsifier of dietary fat, comprises mostly water and cholic acids (chenodeoxycholic, taurocholic, and other derived acids), with up to 5% dissolved salts (including bile salts and bilirubin as anions), organic compounds, vitamin residues, cholesterol, phosphatidylcholine, and heavy metals (such as copper and zinc) [69,70]. As the cutoff for the kidneys is 5.5 nm, larger nanoparticles will be excluded from renal excretion and deposited into the bile for transport out of the body, as will smaller particles that the liver can digest with its diverse array of oxidizing and detoxification enzymes [71]. It has been reported that biliary excretion is the typical elimination route for a small percentage of gold nanoparticles, with the rest sequestered inside nonparenchymal cells resident in the liver [71]. Excretion of silica nanoparticles, conversely, relies on surface charge, since zwitterionic coatings facilitate rapid clearance, while carbon nanotubes larger than the renal cutoff may take several months to clear through the bile duct [72]. Large silica particles (>80 nm) tend to stay resident within the liver but may be desirable due to their large cargo capacity; a recent report asserted that, in such a case, covalent labeling resulted in rapid clearance even of larger particles [72]. Figure 3 summarizes the multiple pathways a nanoparticle may encounter based on its composition.

In patients with hepatic dysfunction (hepatitis, steatohepatosis, cirrhosis, etc.), a reduction in liver function may cause unwanted accumulation of nanoparticles, since bile output may decrease (up to 46% less, as observed clinically) in cirrhotic or fatty livers, while ileal surgery patients (ileostomy) may have defects in bile reabsorption, and changes in bile composition may affect the ability to solubilize lipid nanoparticles [70,73]. On the other hand, cholecystectomy patients lose the ability to concentrate bile, and it continuously drips into the small intestine, which may affect the rate at which lipid nanoparticles are excreted.

#### 2.2.2. Kidneys

The kidneys are essentially an osmotically driven blood filtration system with an effective molecular weight cutoff of 30 to 50 kDa, allowing water and small proteins (<20 kDa) to be excreted freely while retaining albumin (67 kDa) [74]. Particles with sizes of less than 5.5 nm up to 8 nm may also be freely excreted, and thus, smaller-sized nanoparticles may use the kidneys as an important elimination route. Neutrally charged particles may avoid adsorption by larger proteins and facilitate rapid clearance, but damage to the renal tubule network may cause retention of even small particles, and proteinuria from glomerular leakage was not reported to increase gold nanoparticle clearance [75]. As with the liver, zwitterionic-coated inorganic nanoparticles under the 5.5 nm threshold have the fastest clearance, while cationic charges and larger particles tend to be poorly excreted by the renal route [76]. Poor excretion in these cases may cause unwanted bioaccumulation in the kidneys and mechanical damage to the nephron network via scarring and release of plasma-resident, high-molecular-weight proteins (e.g., creatinine) into periglomerular areas, which may cause inflammation and fibrosis [77].

In patients with existing renal dysfunction, cumulative damage to the nephron, as the functional unit of filtration, due to progressive overload and compensation may affect excretion of nanoparticles. Since the osmotic pressure that would ordinarily allow 5.5 nm or smaller particles to diffuse into the urinary tract would not be fully functional, this could lead to bioaccumulation. Such a phenomenon is sometimes seen in renally impaired patients receiving gadolinium contrast agents (in chelated nanoparticle forms of sizes around 6 nm), in which case slow clearance may contribute to renal toxicity [78].

#### 2.2.3. Spleen

The spleen is a large, blood-storing and hematopoietic organ that also functions as a center for immune surveillance and processing, with resident macrophages that may readily take up large (80–100 nm) nanoparticles. These large particles may be partially or fully degraded by lysosomal enzymes, and organic compositions will allow for full degradation, while inorganic remnants may eventually be excreted via the kidneys (if smaller than 5.5 nm) or hepatobiliary ducts (if larger). It is within the red pulp of the spleen that the sieving action responsible for filtering defective erythrocytes may catch and retain larger nanoparticles, while the white pulp contains lymphatic tissue (B-cell and T-cell zones) and immune cells that can sequester particles of all sizes [79]. The splenic parenchyma may thus also be assumed to be exposed to nanoparticles within the blood circulation, and the marginal zone (with a population of specialized metallic macrophages with sialic acid-binding surface molecules) may be an important bioaccumulation point for inert or inorganic particles [79]. This propensity to accumulate larger particles is reported to be beneficial in constructing nanoparticles intended to stimulate or modify immune responses, since bioaccumulation within the spleen exposes the nanoparticle to the entire panoply of T- and B-cell populations [80]. Of course, while splenectomy makes manipulation of the immune response more arduous, at least there will be no concerns regarding unwanted bioaccumulation within the red or white pulp.

#### 2.2.4. Lungs

Inhaled nanoparticle preparations are usually sequestered within the lungs for extended periods of time, as they first contact the mucus layer produced by goblet cells in the epithelium and are taken up by interstitial sentinel macrophages within the lungs for presentation to lymphocytes within the mediastinal lymph node [81]. Low solubility, engineered liposomes, and coatings made via fusion to Fc fragments can further extend the half-lives of nanoparticles delivered to the lungs, while pure proteins may clear rapidly; this may be an important consideration in treating acute vs. chronic conditions [81]. Also of note are the cilia, which beat rhythmically to expel large particles from the lungs that are entangled in the mucus (including larger nanoparticles). As the main rally point for sentinel macrophages, the mediastinal lymph node may serve as a zone of bioaccumulation in cases of inhaled nanoparticle therapies. Murine experiments have indicated that, upon administration of 20 nm gold nanoparticles, approximately 30% are rapidly cleared by the cilia, while, of the remaining particles, 80% shift from the epithelial surface to the interstitial layer within 24 h [82]. These particles then move to the circulation, where they are primarily taken up by the liver, spleen, and kidneys; this indicates that the lungs are not a chief metabolic or excretory route for nanoparticles but facilitate transport to structures better adapted to excrete particulate matter (such as the liver and kidneys) [82]. However, size may play a role in lung disposition of nanoparticles, as large nanoplastic polystyrene particles (100 nm) were found to sequester within cellular mitochondrial compartments, as opposed to smaller (50 nm) particles, which were primarily excreted via lysosomes, in a human lung cell line [83]. In patients with lung diseases, particularly chronic obstructive pulmonary disease, abnormal airflow vortices within the bronchi were shown in simulations to cause hot spots of accumulation of 10–100 nm particles in the upper rather than lower airways, since small size increases the influence of Brownian drag and Saffman lift forces [84]. In smokers, alterations in cilia networks and function may reduce the clearance of nanoparticles, and thus, lung condition is a primary consideration when engineering nanoparticles for medical use in the respiratory/pulmonary system [85].

### 2.3. Clearance Step 2: Cellular Interactions as Barriers to Clearance

#### 2.3.1. Vascular Extravasation and Tumor Penetration

Nanoparticles delivered to tumors must cross an endothelial barrier to gain access to the internal tumor environment; this has been termed the enhanced permeation and retention (EPR) effect, and this extravasation process is highly dependent on the tumor cell type, tumor stage, and “leakiness” of the endothelial barrier. Generally, the permeability of tumors depends on lymph flow, presence and modulus of the extracellular matrix, fluid pressure, and blood vessel permeability [86]. Tumors secrete numerous angiogenic factors (e.g., VEGF), and these blood vessels are often irregularly shaped and constructed, leading to pressure differentials, variable permeability (due to malformation), and higher density, all of which may increase retention through enhanced permeability and reduced perfusion [86]. Additionally, tumor microenvironments (TMEs) are usually low in pH and hypoxic, creating a unique condition that nanoparticles can be engineered to exploit. Thus, inorganic, metallic particles may offer increased EPR by increasing reactive oxygen species (ROS) or regulating angiogenesis, while polymer-based nanoparticles can be created to degrade at pH values usually found in TMEs [86].

#### 2.3.2. Endosomal Escape

Uptake of extracellular materials through endocytotic processes, namely, pinocytosis and phagocytosis (in professional antigen-presenting immune cells), is the general method by which nanoparticles are introduced to the internal cellular environment. Endocytosis traps particles, and these early endosomes are shuttled towards lysosomal fusion by a tightly regulated pathway for digestion of internal cargo and its release into the cytosol. There are diverse endocytotic pathways that rely on transport-specific molecules (flotillin-dependent, clathrin-dependent, RhoA-dependent, etc.); these outnumber the non-endocytic pathways, such as direct fusion and pore formation [87]. Thus, a nanoparticle’s effectiveness and eventual clearance usually depend on escape from these endosomal pathways, and estimates of escape efficiency are quite low (e.g., in the 5–10% range for mRNA nanoparticles). However, increased escape efficiency may increase intracellular inflammation in off-target cells through a galectin-mediated mechanism [87].

Endosomal compartments are formed at the cellular membrane and mature as they deliver cargo through a Rab5-to-Rab7 conversion process, ESCRT-mediated vesicular formation, and V-ATPase acidification of the internal compartment in preparation for LAMP-mediated lysosomal fusion [87]. For this reason, nanoparticle coatings that are pH-controlled could target these endosomal maturation proteins to better facilitate escape, or microbial escape mechanisms (such as pore formation by *Listeria monocytogenes* or membrane destabilization by adenoviruses) could be exploited to enable better movement into the extracellular environment [87]. As such, lipid nanoparticles and other biopolymers may be designed to exploit the pH or aqueous aspects of the intracellular compartment to diffuse drugs after weakening the endosomal membrane with molecules derived from bacterial toxins, wetting agents that swell to burst the membrane, or simple chemicals such as excess calcium [88].

## 3. Properties of Nanoparticles That Control Their Metabolism, Delivery, and Clearance

Nanoparticle properties and modifications that affect their pharmaceutical and biological properties are summarized in Table 2.

### 3.1. Organic vs. Inorganic Persistence: Differences in Hydrolysis

Biodegradable organic nanoparticles (e.g., PLGA/PLA-based systems) normally carry a lower intrinsic risk of bioaccumulation because of their inherent susceptibility to hydrolysis once administered. A report demonstrated this with PLGA nanoparticles, which showed measurable first-order degradation in murine liver, spleen, and lungs [89]. This effect was shown to be reliant on hydrophobicity: 50:50 PLGA nanoparticles completely degraded in vitro in roughly 100 days, whereas an 85:15 hydrophobic-dominant composition only experienced around 60% degradation over the same interval [89]. Thus, the degree of biodegradation depends on the polymers chosen and ratios that affect hydrophobicity, which, in turn, affects exposure burden. However, this host–nanoparticle interaction is also mediated by local acidification, excipients, and potential metabolic context. Chaplin and colleagues intravenously administered PLGA nanoparticles to mice and saw their accumulation within the cecum, which then experienced acidification that altered the microbiome and reprogrammed hepatic transcripts in obese mice; this is despite the polymer being classified as biodegradable [53].

Conversely, inorganic nanoparticles resist elimination through cores that resist oxidation and degradation by somatic enzymes. A report found that BSA-coated 20 nm gold nanoparticles remained lodged in murine organs 120 days after a single intravenous dose, with only a 39% reduction in liver burden but increased splenic (53% increase) and renal (150% increase) burdens. With regard to long-term effects, this persistence was associated with inflammatory and fibrotic responses [90]. While inorganic particles remain susceptible to chemical forces (silica can undergo dissolution under biologically relevant conditions), they are typically resistant to solubility, hydrolysis, and dissolution, which makes composition, porosity, and local microenvironment more important criteria for their persistence compared with organic particles [57,91,92]. Toxic and inflammatory effects may be due to persistence over a long period, but degrading metallic cores may also produce redox stress, as seen with amino-coated CdTe quantum dots, which were shown to readily penetrate cellular membranes but generate enough reactive oxygen species that cell death was observed [93].

**Table 2:** Nanoparticle modifications and impact on pharmaceutically relevant parameters.

Modification Class	Modification Type	Solubility Effect	Cargo Load Effect	Targeting Effect	Immune Effect	Bioaccumulation Effect	References
**PEG; Cholesterol; Hydrophobic/philic Biopolymers**	Micelles	Greatly improves aqueous dispersion of hydrophobic cargo	Medium–high for hydrophobics; low for hydrophilics	Passive targeting common; active targeting if ligand added	Usually decreased opsonization/uptake with PEG; anti-PEG risk exists	Moderate; longer circulation, often RES/liver–spleen if not cleared	[94,95,96]
	Chylomicrons	Increased for lipophilic drugs and oral absorption	High for highly lipophilic cargo	Strong lymphatic/intestinal transport; hepatic lipoprotein pathways	Often low–moderate (biomimetic), but composition-dependent	Moderate–high in lymph/liver; useful when lymphatic delivery is desired	[97,98,99]
	Polysaccharides (alginate, cellulose, dextran, etc.)	Increased for hydrophilic biologics; good colloidal stability	Medium–high; especially useful for genes/proteins and hydrophilic drugs	Can be receptor-targeted (e.g., HA/CD44) and often mucoadhesive	Usually low/holistically compatible, but some polysaccharides are immunostimulatory	Low–moderate if biodegradable; RES uptake rises with larger/charged particles	[100,101,102]
	Proteins (Zein, Casein, etc.)	Medium; often helps stabilize sensitive actives more than it raises solubility	Medium–high for hydrophobics and bioactives	Good surface functionalization; some protein interactions aid uptake	Low–moderate; holistically compatible but antigenicity can occur	Usually low–moderate because proteins are biodegradable; formulation matters	[103,104]
	PLA, PCL, PGA	Increased apparent solubility/bioavailability of poorly soluble drugs	High for hydrophobic small molecules; sustained release common	Mostly passive unless ligands are added	Usually low, but acidic degradation products/surface charge can irritate	Moderate; often liver/spleen RES deposition unless stealth-coated	[105,106]
**Ionic**	Ion pairs	Water solubility of free drug often lower, but carrier compatibility/partitioning increased	Increased for charged small molecules; otherwise hard to encapsulate	Little intrinsic targeting; mainly an encapsulation strategy	Usually neutral; immune profile driven by the final carrier	No strong intrinsic effect; depends on carrier and release kinetics	[107,108]
	Crystalline core	Often neutral or lower dissolution rate, but physical stability rises	Very high possible in drug nanocrystals/crystalline cores	Mostly passive unless surface-modified	Usually low from matrix itself; surface can still opsonize	Depends strongly on size/coating; can increase residence as depot particles	[109,110,111]
	Covalent linking	Tunable: can increase with hydrophilic linker or decrease with hydrophobic conjugate	Moderate and stoichiometric; highly reproducible loading	High when using cleavable prodrugs/attached ligands	Can mask epitopes or create new ones; chemistry-dependent	Often higher circulation and target-tissue retention; risk of retention if linker is not cleaved	[112,113,114]
**Surface Molecules; Mimics**	Enzymatic substrate	Little direct effect on bulk solubility	Medium	High in tissues rich in the trigger enzyme	Can reduce systemic exposure; may activate local immune effects after cleavage	Usually lower off-target accumulation, higher at enzyme-rich lesions	[115,116]
	pH-responsive	Triggered release/charge-switching at target pH often higher effective local solubility	Medium–high	High for acidic endosomes/tumor microenvironments	Can enhance local immunotherapy while reducing systemic immune exposure	Usually less off-target release; more retention where pH trigger exists	[117,118]
	Heat-responsive	Thermal trigger can switch hydration/solubility and accelerate release	Medium	Moderate–high when external heat or hyperthermia is available	Often synergizes with hyperthermia-induced immune activation	Localized retention/release at heated site; otherwise depends on carrier	[119,120,121]
	CD mimics	Greatly increased for poorly soluble guest molecules via inclusion chemistry	Medium–high for compatible small molecules	Low intrinsic targeting; high if further functionalized	Usually low, but modified CDs can have toxicity/immunologic issues	Usually low–moderate; small systems may clear renally, larger ones follow RES	[122,123,124]
	Ligands (peptides, etc.)	Little direct effect on solubility (unless ligand is strongly hydrophilic/hydrophobic)	Slightly low to neutral due to surface occupancy	Greatly increased receptor-specific uptake and cell selectivity	Can increase opsonization or immunogenicity; stealth spacers help	Higher target-tissue accumulation when receptor is present; off-target RES still possible	[125,126,127]
**Size**	2–5 nm	High colloidal dispersibility possible	Very low	Excellent tissue penetration, but limited payload and short residence	Often lower macrophage uptake; surface chemistry still matters	Low overall due to rapid renal clearance (<~5.5 nm)	[128,129]
	5.5–10 nm	High–medium	Low–medium	Good balance of penetration vs. residence; near renal threshold	Low–moderate; smaller = better Th1 and Th2 responses	Low–moderate; partial renal clearance still possible near ~5.5–6 nm cutoff	[128,130,131]
	10+ nm	Medium	Medium–high	Often better payload and multivalency; passive tumor uptake possible	Moderate–high opsonization/RES risk as size rises	Moderate–high in liver/spleen and other RES organs	[129,132,133]
	µm size	Suspensions possible, lose nano-sized properties	Very high	Best for local, depot, inhaled, oral, or phagocyte-directed use; poor deep tissue penetration IV	High phagocytic uptake/inflammatory risk	High local retention or macrophage capture; embolic risk if injected IV	[133,134]
**Shape**	Rod	Less soluble than spheres	Holds more cargo than spheres	Better at tumor infiltration	Internalized better by professional antigen-presenting cells; stronger Th2 response	Higher retention than spherical particles, especially in liver and kidneys	[131,135,136]
	Spherical	Can be controlled with micellar modification	Basic cargo capacity	The targeting standard by which other shapes are measured	Stronger Th1 response	The retention standard by which other shapes are measured	[131,135,136,137]

### 3.2. Surface Modification and Size

The surface of the nanoparticle, as the chief interface between the particle and cellular environment, forms the corona from proteins and biological molecules that interface with and adsorb to the particle. These coronas may be soft (loosely bound) or hard (tightly bound) and change the surface charge, molecular weight, size, and potentially the targeting capacity of the particle. These changes may even affect the ability to pass through biological barriers that exclude molecules based on charge, size, hydrophobicity and other criteria (such as the blood–brain barrier) [138]. Thus, even with the specific surface modifications described below, the impact of the dynamic and highly variable coronal formation on pharmaceutically relevant parameters must be fully elucidated before use [139].

Dynamic protein corona formation can significantly alter both passive and active nanoparticle targeting because the newly formed, adsorbed protein layer, rather than the original engineered surface, may cause changes in size, surface presentation, immune recognition, solubility, circulation time, and tissue distribution [140,141]. For active targeting, the major risk is masking of the ligand or unwanted steric/biochemical interference since protein corona formation has been shown to significantly reduce the effective targeting yield of ligand-coated nanoparticles [142]. Using the blood–brain barrier (BBB) as an example, this problem becomes especially acute since receptor-mediated transcytosis (crossing of the barrier) is limited by capacity and is also highly selective. A report by Xiao and colleagues demonstrated that transferrin-modified, PEGylated polystyrene nanoparticles had attenuated transcytosis (uptake and release by the barrier cells) with the in vitro corona versus the in vivo corona [143]. Moreover, the corona is not static during BBB transit and may undergo modification during the endocytosis, intracellular trafficking, and exocytosis processes required during transcytosis [143]. This means that even the pre-BBB in vivo corona does not reliably predict the protein structure of the post-BBB corona [138]. However, this process can also be exploited since apolipoprotein- or vitronectin-enriched coronas are more readily transported across the BBB; thus, corona engineering may be an important experimental parameter in nanosystem designs targeting the CNS [144,145].

A. PEGylation or cholesterol

Polyethylene glycol is a safe, bio-inert polymer often used to coat nanoparticles to both evade immunogenic reactions and resist digestion to extend blood persistence. However, this coating is also amenable to opsonization, in which antibody fragments are used to specifically target nanoparticles to APCs for phagocytosis, and it has a hydrophilic nature that aids in solubility [8]. Additionally, PEG molecules can be used to construct a large cargo area of high molecular weight (2 to 20 kDa) that prohibits penetration of foreign particles and, unless opsonized, does not readily interact with immune cells or other proteins if the coating is over 2 kDa [8]. The utility of PEG also changes with concentration, as aggregation and shielding properties depend on the percentage of PEG in the overall nanoparticle composition, and conformational structures are also a function of PEG content [8]. Other biopolymers, such as dextran (polysaccharide), polydopamine (from shellfish), hyaluronic acid, or chitosan, may be used instead of PEG to avoid protein adsorption or opsonization with circulating antibodies [146,147]. These modifications to increase penetration extend the useful half-life in nanoparticles, and this phenomenon is known as the EPR [148].

Cholesterol coatings are useful for encapsulating mRNA for delivery because they easily pass through cell membranes, and the hydrophobic nature of cholesterol stabilizes the ribonucleic payload and ionizable lipid component of some nanoparticles [149]. Cholesterol can also be integrated with other components (such as PEG, lipids/phospholipids, etc.) and can passively defend the nanoparticle against random protein adsorption [149]. A recent study proposed that the synthetic Mycobacterial monomycoloyl glycerol analog MMG-1 functions similarly to cholesterol in mRNA nanoparticles but is superior at stabilizing the lipid membrane component [149]. Thus, the use of coatings with engineered hydrophobicity or hydrophilicity, such as PEG or cholesterol, may be important for both persistence and targeted delivery, but maintaining shorter polymer chains and neutral charges, in addition to strong hydrophilicity, will ensure quicker clearance, while larger polymer chains, positive charges, and hydrophobic coatings will lead to persistence within the body, increasing EPR, even if many particles end up in professional phagocytes.

B. Zwitterions, polysarcosine, and poly(2-oxazoline) coatings

Zwitterions (dipolar ions) have positively and negatively charged functional groups but a net charge of zero. This property allows for specific targeting, as well as affecting adsorption by somatic proteins. For example, the hydrophobic mucus barrier within the small intestine is negatively charged, and a zwitterion, with an overall neutral charge, may more easily slip through, especially when combined with a hydrophilic molecule like PEG [150]. This overall neutral charge/hydrophilic combo can also act as a stealth mechanism to evade immunogenic reactions. Zwitterions have some additional promise for self-assembling nanoparticle purposes, since the functional groups can easily promote secondary and tertiary conformational changes within overall amphiphilic moieties [151]. The overall neutral charge may also better facilitate renal excretion.

Polysarcosine is a polymerized, hydrophilic version of sarcosine (N-methylated glycine) that exists endogenously and exhibits immune-evading properties that make it useful for furtive coatings of mRNA delivery nanomedicines [152]. Like most polymerized coatings, the chain length and concentration of polysarcosine control the physicochemical properties of the coating, while complexes with other components (e.g., ionizable lipids, cholesterol, etc.) allow for fine-tuning of the nanostructure to meet delivery requirements [152].

Poly(2-oxazoline) is a polymer class comprising tertiary polyamides that are derived from polypeptides and function as structural isomers [153]. These polymers have a monomer side chain that can be modified with carbon chains to create diverse capabilities based on functional group size and type [153]. Thiols, esters, amines, halides, and other groups are all possibilities and are sequentially added in an initiator–monomer–terminator arrangement [153]. These coatings may also be used to control the excretion/elimination rate by engineering specific charge or hydrophobicity parameters.

C. CD mimics (e.g., CD47), proteins/peptides, and carbon dot nanozymes

Nanoparticles may have protein moieties attached to their surfaces (peptide-based nanoparticles), and these may be derived from cluster of differentiation (CD) cell surface or other diverse proteins. These sequences of amino acids may be 2–50 residues and are being investigated, as they come from biological backgrounds and are thus highly compatible for pharmaceutical development [154]. A recent example is CD47-cloaked lipid nanoparticles that are coated with the APC CD47 surface protein, which prevents phagocytosis; these particles were shown to successfully evade immune engulfment and deliver an mRNA payload to hematopoietic stem cells via surface targeting with opsonized antibody fragments [24]. Another group demonstrated that designed ankyrin repeat proteins (DARPins) can be bound to nanoparticles (along with other proteins, such as ApoE) instead of antibodies for specific targeting [155]. Since ankyrin serves to anchor membrane proteins, they consist of a repeated domain that can be replicated easily by bacteria to generate large amounts of DARPins [155]. The versatility of peptides also extends to nanogels that can be synthesized from extracted peptides, Tween wetting agents, and other compounds (like DMSO) required to solubilize the payload; a recent report used an Nα-9-fluorenylmethoxycarbonyl-diphenylalanine (Fmoc-FF) peptide-based nanogel to deliver doxorubicin and curcumin to thyroid cancer cells, finding that the peptide-based nanogel maintained extended drug release [156]. Furthermore, nanoparticles derived from functional body proteins, such as elastin, have been shown to be amenable to chain length modifications that, for example, increase their capacity to force cadmium out of an aqueous solution into a colloid suspension [157].

Peptide-based nanosystems thus have vast potential due to their customizable nature but may be inherently limited by their very biocompatible origins since proteases and other enzymes may prematurely react and damage the attached amino acids, while heat and reactive oxygen species that affect binding may also limit persistence and on-target delivery [154]. Nonetheless, this low persistence may be of value if rapid clearance is desired, and the high holistic compatibility may address long-term deposition concerns.

Other biological molecules that may be less immunogenic and more specific for therapy targeting (up to 2–10 × more specificity) are aptamers (short oligo chains of ssRNA or ssDNA), which can be added externally to nanoparticles. These particles require no cargo capacity, can be made extremely small, can exploit strong and specific binding to DNA or can even deliver siRNA to specific targets [158]. Aptamers are attractive because they are compatible with diverse ranges of nanoparticle raw materials (gold, carbon, silica, quantum dots, etc.), but they are also highly susceptible to forces (pH, enzymes, reactive oxygen species) that degrade nucleic and ribonucleic acids [158]. While this also limits the functional half-life of such particles, it may be desirable, as it allows for more rapid clearance after therapy.

Carbon dot nanozymes are artificial, nano-sized (10 nm), inorganic enzymes that catalyze biochemical reactions based on elements (such as iron, copper, or manganese) used to dope the carbon [159]. These nanozymes can mimic superoxide dismutase, dehydrogenase, and other metal-based enzymes within the body while featuring low toxicity and good biocompatibility/clearance [159]. There are also carbon nanozymes derived from decomposed biomass (herbs, broccoli, coffee, etc.) that retain functional groups from their original forms, such as carboxyl, amino, or sulfate groups, that allow for various capabilities; these nanoparticles may also undergo covalent doping with metals for additional functionality [159]. However, inorganic particles do carry inherent risks for incomplete metabolism, bioaccumulation, off-target activity, and long-term safety. Cytotoxicity risks due to accumulation are a chief concern, and carbon dots have been demonstrated to have LD50 amounts as low as 100 ppm in fish embryos, even if lower concentrations of dots were cleared quickly [160,161]. Additionally, organic particles that are resistant to oxidation in the liver or by lysozymes (silica, gold) may persist in endosomes, generating oxidative species, inducing inflammation, and creating genotoxicity [162]. Off-target accumulation is also a serious issue, as inorganic particles tend to be taken up by phagocytes and may accumulate in the liver (hepatotoxic) and spleen unless guided to specific targets by engineered coatings [161,163]. Collectively, these issues and the lack of clinically relevant long-term safety data make the use of inorganic nanoparticles a field that requires further development, and the use of biomimetic compounds (such as bacterial or cell-sourced coatings) that prevent unwanted accumulation and off-target action may increase long-term safety [161].

In addition to opsonized antibodies and peptides (such as transferrin), low-molecular-weight molecules like folate are possible and have been investigated in the targeting of folate receptors on tumor cells with iron oxide nanoparticles during magnetic hyperthermia treatment [164]. These molecules are applied to a dextran coating, but dipeptide gel coatings are also frequently exploited for their low molecular weight and biocompatibility [147,164].

D. Size and Shape (deliberate exclusion)

As the cutoff for renal filtration is 5.5 nm, larger particles may be retained longer and accumulate better at target sites but be selectively cleared much faster by the immune system [165]. Conversely, smaller particles (2–10 nm) would feature lower accumulation at the target and have higher passive clearance but may be harder to clear from targeted sites once captured within cells [165]. Larger-sized particles have a greater cargo capacity and space for external functional groups as well, but these may interfere with targeting or mark the particle for clearance by professional phagocytes, while smaller particles may evade the immune system better but have less room for cargo and external modification. Shape is also a concern, as several reports have demonstrated that rod-shaped nanoparticles have better retention and solubility than spherical particles. For immunogenicity, spheres are useful for Th1 responses, while rods are better at evoking Th2 responses [131]. Thus, both shape and size are important considerations that affect final clearance.

### 3.3. Nanoparticle Features That Affect Payload Distribution and Rate of Distribution

A. Micelles

Micelles are amphiphilic, often polymeric, and self-assembling, with a convenient hydrophobic core and hydrophilic outer shell. Characteristics like pH sensitivity, enzyme reactivity, and temperature response make them excellent for dispersing cargo from nanoparticles [166]. The rate of dispersal can be controlled by adding linkers or coatings that resist or facilitate response to the microenvironment that the micelles are designed for. Unlike chylomicrons, which are lipoprotein-based and coated with proteins, micelles are readily absorbed and reduced to triglycerides once their cargo is delivered, increasing their holistic compatibility through an existing clearance system. Chylomicrons, on the other hand, transport large lipids through the lymph, where they can be hydrolyzed by lipases to release cargo [167]. Thus, unless the lymph system is the target, micelles are superior to chylomicrons for cargo delivery through the bloodstream and for better clearance after delivery.

B. Ion pairs/crystalline core

A core configuration based on ion pairs is usually seen in inorganic particles (silica, zinc, etc.); these can theoretically load hydrophobic cargo, but the addition of such molecules to an ordered core will eventually disrupt the core due to molecular forces, and disassembly is a frequent result [168]. However, strategies to maintain core integrity, like semi-crystalline polymers, as reported by Ganda and colleagues with their glycopolymer system exploiting poly(ε-caprolactone)-b-poly(1-O-acryloyl-β-d-fructopyranose) (PCL-b-PF), may allow for non-disruptive cargo loading, as the polymer will continually resist disassembly through spontaneous self-assembly due to internal molecular forces [168]. Nanocrystals composed entirely of the therapeutic drug are also in development, and these are reputed to extend stability in vivo through stabilization of hydrophobicity and charge; they are also amenable to surface modification with ligands (e.g., folic acid or hyaluronic acid) [111]. Such considerations are important for engineering the functional half-lives of such core–shell-based nanoparticles and also for clearance after therapy.

Cargo retention, in vivo stability, and clearance are also likely to be affected by ionic or crystalline cores since net charge and hydrophobicity are key aspects in resisting premature disassembly and fragmentation. Within the kidney, the double glomerular barrier system uses size and charge as the primary criteria to exclude particles, and nanocrystals with a net neutral or positive charge may pass more easily, while a net negative charge would ablate excretion through the kidneys and allow for bioaccumulation [5]. In this case, highly negative charges and increased hydrophobicity on the outer particle surface could be important modifications for particles intended for kidney retention (e.g., for kidney cancer).

C. Covalent linkers

Covalent binding forces can be exploited to create “superlatticed” structures of specific sizes comprising metallic nanoparticles [169]. Three-dimensional structures are possible through mercaptohexanol-prepared gold nanoparticles, and esterification reactions can add functional ligands for interactions between nanoparticles within the superlattice [169]. While these covalently linked lattice structures may be too large for current internal applications, they may be useful in future dermatological or external detection applications (e.g., a skin cream that reacts with a color change when it detects melanoma)

D. Enzymatic substrates, pH-responsive, and heat-responsive elements

Enzyme-reactive surface coatings, pH-sensitive cargo compartments, and heat-responsive elements are reliable and important mediators of cargo release since all three can be tested extensively in vitro to detail their response dynamics. Since pH is well-controlled within the body but is often acidic in tumor microenvironments and other specific spots (e.g., the stomach, lysosomes), construction of pH-sensitive nanoparticles using polylactic-co-glycolic acid and albumin designed to penetrate tumor cells and then deliver cargo after lysosomal processing has been reported [170]. Enzymatic reactions also proceed in a pH-dependent fashion, and functional groups on the nanoparticle’s exterior may become active at specific pH values, while cargo release can be accomplished in systems based on the microenvironment. Lui and colleagues engineered such a system using ferritin that released an anti-PD-L1 peptide based on acidification by lysosomes within tumors as well as matrix metalloproteinases [171]. These pH-responsive and enzyme-conjugated nanoparticles have very little, if any, leakage before processing, making them useful in targeting tumor cells with high concentrations of therapeutic cargo. Similarly, temperature-sensitive nanoparticles may be useful in concert with hyperthermia or cryotherapy and a phospholipid system capable of a gel-to-liquid crystalline phase shift that affects the permeability of the bilayer [148]. Such systems exploit this reversible property of phospholipids to deploy functional groups since gel-state phospholipids have a compact configuration with packed side chains, and the liquid-state shift allows the chains to deploy within specific temperature ranges (e.g., 30–38 °C) [148]. This release allows for bioaccumulation in target areas and then rapid deployment of cargo and subsequent clearance.

E. Ligand density optimization

A critical aspect of payload distribution is ligand-based targeting, in which short oligonucleotide sequences or RNA aptamers are attached to nanoparticles to bind to cellular surface receptors specific to the targeted cell, which can be a somatic, immune, or tumor type. However, the optimal surface-area-to-ligand density and surfactant properties must be optimized to the target; for example, Fakhari and colleagues found that the optimal density of an ICAM-1-binding cyclic peptide on PLGA nanoparticles with and without coumarin-6 was achieved with a 50:50 or 25:75 ratio of Pluronic F68:Pluronic F108 [172]. This is due not only to binding interference/steric hindrance based on ligand density but also to the alteration of particle charge and size by conjugation of surfactant molecules bound to the nanoparticles themselves [172]. Another report by Zhang and colleagues on mannose-modified trimethyl chitosan–cysteine nanoparticles stated that lower concentrations of mannose (4%, as opposed to 13% or 31%) have higher macrophage uptakes, most likely due to molecular weight, while Shen and colleagues reported an optimal uptake density of 1:10 (wheat germ agglutinin: maleimide) in PEG-PLA nanoparticles targeting Calu-3 cells [173,174]. These data, among various other reports, collectively indicate that molecular weight, steric hindrance, and uptake ability are heavily weighted by ligand density, and any engineered nanoparticle that exploits ligand binding will require optimization to determine the best ratio of ligand to surface area.

F. Receptor-mediated uptake efficiency

Another consideration in payload distribution is the efficient uptake of nanoparticles by the intended cell-surface receptor, as these processes are energy-dependent and tightly regulated in both normal and transformed cells. While protein coatings have been shown to activate endocytosis, modified binding proteins, such as apoferritin, were previously demonstrated to allow for more efficient uptake of platinum nanoparticles, as ferritin receptors are found on virtually every cell type [175]. Another report by Liu and colleagues indicated that polydopamine-coated gold nanoparticles could readily interact with surface dopamine receptors on Kupffer cells for better uptake [176]. In tumor cells, which highly express folate receptors (up to 500 × more than non-tumor cells), gadolinium–folate tracers have been shown to display high affinity for bioaccumulation in solid tumors, and reports of iron oxide–folate nanoparticles have shown promise for magnetic resonance imaging [177]. These receptors, outside of target specificity, are therefore reliable and efficient methods for high uptake of nanoparticles.

G. Off-target binding

A chief concern of nanotherapy and a primary driver of ligand/receptor studies in nanoparticle target specificity is off-target binding. Inefficient delivery of the therapeutic agent may occur if targets are found in a wide distribution of cell types, if intracellular processes or hepatic detoxification enzymes modify or digest nanoparticle coatings, or if particles cross endothelial barriers errantly. Thus, much effort has been made to modify natural protein-derived coatings (such as using cationic lipid 1,2-dioleoyl-3-trimethylammonium propane [DOTAP] and DNA together to enrich protein diversity into the hundreds while increasing vitronectin amounts to 30% of total protein) or test multiple variants of RNA aptamer sequences to maximize specificity and reduce off-target binding [178]. In general, targets are chosen based on relative abundance in the targeted cell type (e.g., folate receptors in tumor cells) [177]. It is also important to test multiple variants of micromolecules, especially RNA-based oligonucleotides, as was shown in a report by Parrett and colleagues, in which the number of binding sites in 5′ and 3′ untranslated regions of luciferase were important in determining the binding efficiency of miR-122 [179]. Thus, receptor targets with fewer miRNA binding sites may lower targeted binding, and other receptors on undesired cell types may instead accumulate such nanoparticles. If serum-bound proteins are used, it is advisable to enrich for species that increase desired binding since albumin is reactive and blocks binding, while serum may contain hundreds of other protein components that could possibly interfere [180]. Taken together, off-target binding is a key aspect of nanoparticle engineering and development of cell-specific ligands is expected to be an area of constant growth in translational research.

## 4. Reconsidering Nanoparticle Clearance: Turning a Limitation into a Therapeutic Advantage

### 4.1. Clearance Is Not Always a Failure Parameter

As we discussed previously, in classical pharmacokinetics, rapid clearance is typically regarded as a desirable safety feature, minimizing systemic toxicity and off-target accumulation [181]. Based on this concept, nanomedicine design has historically prioritized stealth properties, renal excretion, and biodegradability to avoid long-term retention in the body. However, this paradigm does not universally apply to all therapeutic modalities. For treatment strategies in which therapeutic efficacy depends on prolonged intracellular or intratumoral presence, rapid clearance represents not an advantage but a fundamental limitation. This is particularly evident in boron neutron capture therapy (BNCT) and may extend to other radiation-based or energy-dependent therapies where sustained target loading is essential for therapeutic success, making controlled intratumoral retention a distinct therapeutic advantage [182]. Importantly, however, prolonged retention must be carefully controlled, as excessive or non-selective persistence may lead to off-target accumulation and long-term toxicity in normal tissues.

### 4.2. BNCT as a Model System Highlighting the Limitations of Rapid Clearance

BNCT is a binary radiation therapy modality that relies on the nuclear reaction between thermal neutrons and the stable isotope boron-10 (^10^B), producing high-linear-energy-transfer (high-LET) α-particles and lithium nuclei with a tissue penetration range of approximately 5–9 µm, comparable to the diameter of a single cell [183]. This reaction produces radiation damage to tumor-cell DNA, spatially confined to only boron-containing cells, while surrounding boron-free tissue is largely spared [184]. Therapeutic efficacy, therefore, critically depends on achieving and maintaining sufficiently high intracellular concentrations of ^10^B within tumor cells at the time of neutron irradiation, as the radiation effect is strictly proportional to the local boron content present during exposure. This unique feature makes BNCT exquisitely dependent on intracellular boron concentration at the time of neutron irradiation. Currently, the clinically approved boron carrier boronophenylalanine (BPA) demonstrates rapid cellular uptake via amino acid transporters, but also rapid efflux, resulting in limited intracellular retention [182,185]. Consequently, clinical BNCT protocols require continuous or prolonged intravenous infusion of BPA throughout neutron irradiation, which may last one hour or longer. This pharmacokinetic mismatch between boron residence time and irradiation duration is a recognized limitation of current BNCT agents.

### 4.3. Prolonged Intratumoral Retention as a Route to Therapeutic Amplification

If boron-containing nanoparticles could be retained within tumor tissue for extended periods, several therapeutic advantages would emerge, including (1) extended irradiation windows, decoupling boron delivery from neutron exposure timing; (2) repeated or fractionated neutron irradiation without re-administration; (3) higher effective tumor dose without increasing systemic exposure; and (4) potential synergy with other radiotherapeutic modalities. Such an approach transforms clearance resistance into a dose-amplifying mechanism rather than a toxicity risk, provided that tumor selectivity is maintained.

This concept is consistent with established observations of nanoparticle retention in tumors via the EPR effect [186] but extends it by emphasizing intracellular immobilization, not merely vascular or interstitial accumulation [187].

### 4.4. Intracellular Enzymatic Self-Assembly as a Retention Strategy

A rapidly expanding body of evidence demonstrates that enzyme-instructed intracellular self-assembly (EISA) represents an effective strategy for enhancing intracellular retention of therapeutic agents by converting small, diffusible molecular precursors into supramolecular nanostructures after cellular entry. In this approach, low-molecular-weight compounds freely penetrate cell membranes and subsequently undergo enzymatically triggered transformations, which are most commonly dephosphorylation by alkaline phosphatases, esterase-mediated cleavage, or protease-activated reactions, leading to the spontaneous formation of higher-order assemblies within the cytoplasm or specific subcellular compartments. These intracellularly assembled nanostructures show reduced diffusivity and are therefore resistant to cellular efflux mechanisms and metabolic clearance, resulting in prolonged intracellular residence. Such enzyme-triggered self-assembly systems have been extensively investigated in oncology as a means to extend intracellular drug activity, enhance tumor selectivity, and induce localized cytotoxicity while minimizing systemic exposure [188,189]. In the context of BNCT, this paradigm suggests that boron-containing agents could be delivered as small, rapidly internalized molecular precursors that subsequently undergo enzyme-driven intracellular self-assembly within tumor cells, thereby substantially increasing intracellular boron retention during neutron irradiation and overcoming the limitations associated with rapid efflux of conventional boron carriers.

### 4.5. Polymer Self-Assembly and Intracellular Immobilization of Nanoparticles

Polymer-based nanoparticles offer an additional strategy for prolonging intracellular retention beyond small-molecule enzyme-instructed self-assembly systems, as they can be designed to undergo secondary self-assembly or cross-linking reactions (e.g., micelle formation, sialic acid) after internalization by tumor cells [190]. These intracellular transformations are typically triggered by tumor-associated microenvironmental cues, including lysosomal acidification, intracellular redox gradients, and the cleavage of enzyme-sensitive linkers, resulting in the formation of polymeric networks or cross-linked assemblies within the cytoplasm or intracellular organelles. Such in situ polymer restructuring markedly reduces nanoparticle mobility and suppresses intracellular efflux, thereby limiting clearance and extending residence time at the therapeutic site. This approach has been shown to significantly enhance intratumoral retention and therapeutic efficacy in multiple preclinical cancer models. For example, Shen et al. demonstrated that therapeutics capable of stimulus-responsive intracellular polymerization formed stabilized assemblies within tumor cells, leading to reduced cellular efflux, prolonged retention, and improved antitumor performance [191].

### 4.6. Implications Beyond BNCT

Although boron neutron capture therapy (BNCT) provides a particularly illustrative example of therapeutic efficacy being limited by rapid clearance of active agents, the same underlying principle extends to a broader range of treatment modalities that depend on sustained intratumoral or intracellular presence of functional components. These include radiosensitizing nanoparticles (often inorganic, such as silica- or carbon-based systems) used to amplify the effects of external beam radiation, photothermal and photodynamic therapies that require prolonged retention of photoactive agents within tumor tissue, and other high-LET particle therapies in which therapeutic outcome is directly linked to the local concentration and residence time of sensitizers at the target site. In such contexts, deliberately slowed clearance and prolonged retention can enhance the therapeutic index when combined with spatially confined or externally triggered activation, provided that off-target accumulation is minimized.

At the same time, prolonged retention raises important safety concerns that must be critically addressed. While increased intracellular or intratumoral persistence can enhance therapeutic efficacy in activation-dependent systems, excessive or non-selective retention may result in accumulation in normal tissues and organs involved in nanoparticle clearance, including the liver, spleen, and kidneys. In the specific context of BNCT, elevated boron concentrations are beneficial only when selectively localized within tumor cells; the presence of boron in normal tissues at the time of neutron irradiation may lead to localized, high-LET radiation damage. Therefore, the objective is not maximal persistence itself but controlled, target-restricted retention with eventual clearance. Strategies to achieve this balance include the use of biodegradable or metabolically cleavable materials, stimulus-responsive systems that promote retention preferentially in tumor microenvironments, intracellular self-assembly approaches, and physicochemical optimization to enable delayed but complete elimination after therapeutic action. This concept is supported by general reviews highlighting the critical role of nanoparticle biodistribution, retention, and clearance kinetics in determining therapeutic performance across multiple nanomedicine-based treatment strategies [192,193].

Taken together, the findings discussed above support a conceptual reframing of nanoparticle clearance from a universal optimization goal to a context-dependent design parameter. While rapid clearance remains a desirable and often necessary feature for systemically acting drugs to minimize off-target exposure and long-term toxicity, controlled persistence and delayed clearance can be advantageous for therapeutic modalities that rely on energy-dependent or spatially confined activation mechanisms. In such cases, prolonged intratumoral or intracellular retention enhances the effective therapeutic window without proportionally increasing systemic burden. In the specific context of BNCT, the intentional engineering of nanoparticles to achieve sustained intracellular retention within tumor cells represents one of the most promising strategies for next-generation boron delivery systems. This approach enables improved temporal alignment between boron localization and neutron irradiation, ultimately maximizing therapeutic efficacy.

## 5. Future Directions

As nanoparticle development shifts into more individualized and goal-specific therapeutic design, future progress will also pivot towards engineering platforms that can control both targeting and drug release, as well as biodistribution, persistence, and clearance, with greater and more reliable precision. Several of these strategies already have experimental or preclinical support, including stimulus-responsive systems, polymer-based carriers with tunable degradation profiles, and surface modifications designed to reduce opsonization or prolong circulation. Triggered systems, such as responsive nanoparticles (magnetic-, pH-, or light-sensitive), are particularly promising because they may allow spatiotemporal control over cargo release after accumulation at the target site. Likewise, natural polymers and other holistically compatible materials, including carbohydrate-derived or zwitterion-coated formulations, may offer improved control over degradation kinetics, immune recognition, and excretion behavior.

At the same time, some future possibilities remain more speculative and are longer-term engineering goals instead of established options. These include multi-functional platforms designed to act sequentially across organ systems or broadly adaptable carrier architectures that are customizable across drug classes. For such approaches to become clinically realistic, persistent translational barriers (including reproducible manufacturing, predictable biodistribution, avoidance of long-term tissue retention, and maintenance of safety across repeated dosing) remain barriers to be overcome. Thus, the next phase of nanoengineering will likely be defined not simply by more complex designs but by matching clearance and persistence profiles to specific therapeutic objectives while preserving clinical and financial practicality.

## 6. Conclusions

In summary, nanoparticle clearance is a central and clinically critical design parameter that shapes safety, efficacy, and translatability across therapeutic applications. Nanomedicines are governed by interconnected micro-level processes, including enzymatic degradation, pH responsiveness, solubility behavior, and immune interaction, as well as macro-level clearance pathways involving the hepatobiliary system, renal filtration, splenic sequestration, and pulmonary handling. Together with individual variability among patients, these processes create distinct clinical constraints that can be modulated through particle size, charge, surface chemistry, and functional coatings.

Importantly, the optimal clearance profile is not universal. Rapid elimination remains desirable when minimizing off-target exposure, and long-term toxicity is the dominant priority, whereas delayed clearance or controlled intracellular persistence may be advantageous when efficacy depends on prolonged retention (as in BNCT). The most effective future nanomedicines will therefore exceed foundational specifications (e.g., circulate longer or clear faster) and have biodistribution and clearance kinetics that are specifically aligned with the intended therapeutic mechanism. In this sense, clearance should be viewed not as a passive consequence of nanoparticle design but as an active and tunable determinant of clinical performance.

## Figures and Tables

**Table 1:** Representative studies of nanoparticles for delivery of therapeutics. PLGA = Poly(lactic-co-glycolic acid); PEG = Polyethylene glycol; PLA = Polylactic acid.

NP Type	Year	Author	Construction	Target	Therapy	Ref
**Lipid**	2024	da Silva	Ionizable lipid	Colon cancer	RNA	[13]
	2024	Jiang	Ionizable lipid	IL-2	RNA	[14]
	2023	Zeng	Ovalbumin	T-cell receptor	RNA	[15]
	2023	Douka	Ionizable lipid	NK cells	RNA	[16]
	2022	Ferraresso	Ionizable lipid	Factor VII	RNA	[17]
	2022	Johnson	Lipid plus APOE binder	Liver cancer	RNA	[18]
**PLGA**	2024	Liang	PLGA/PEG	SOAT2	RNA	[19]
	2023	Ma	PLGA	Chondrocytes	Rapamycin	[20]
	2021	Kim	PLGA	Cochlea	Dexamethasone	[21]
	2020	Jo	PLGA	Macrophages	CRISPR	[22]
	2018	Son	PLGA–folate	Gastric cancer	Pheophorbide A	[23]
**Biopolymer**	2025	Papp	Peptide coating	CD47	Immune evasion/mRNA delivery	[24]
	2022	Chen	Chitosan	Breast cancer	Dox/cinnemaldehyde	[25]
	2021	Sankar	Peptide	Bleeding control	RADA16 self-assembling β sheet	[26]
	2020	Huo	Dextran	Skin cancer	Silybin-Paclitaxel	[27]
	2016	Foerster	Dextra	Liver myeloid cells	RNA	[28]
	2013	Ganesh	Hyaluronic acid	Lung tumor	RNA/cisplatin	[29]
	2013	Ryan	Chitosan	NRA42	Calcitonin–hyaluronic acid	[30]
**Inorganic**	2023	Simón	Gold	Colorectal cancer	Doxorubicin	[31]
	2019	Rastinehad	Gold	Prostate tumor	Gold	[32]
	2018	Guisasola	Silica-FeOx	Lymphoma	Doxorubicin	[33]
	2012	Lu	Silica-Folate	Pancreatic cancer	Campothecin	[34]
	2009	Bhirde	Carbon tube	Skin cancer	EGF-Cisplatin	[35]
**PLAs**	2023	Gao	PLA	Dendritic cells	Carboxyl/Ester Groups	[36]
	2018	Ghasemi	mPEG-PLA	Sustained systemic release	rhGH	[37]
	2010	Primard	PLA	Immune cells	Vaccine	[38]
	2009	Rancan	PLA	Dermal drug delivery	Fluorescent dye	[39]
	2007	Dong	MPEG-PLA	Breast cancer	Paclitaxel	[40]
**Hygroscopes/Bile Derivatives**	2024	Mohsen	Bilosome	Kidney disease	Rutin	[41]
	2023	Soliman	Lactoferrin bilosome	Diabetes	Qurcetin	[42]
	2016	Huang	Polysorbate 80	Brain barrier	Drug delivery	[43]
	2013	Wilkhu	Bilosome from cholesterols	Peyer’s patches	Vaccine	[44]
	2004	Sun	Polysorbate 80	Brain barrier	Drug delivery	[45]

## Data Availability

No new data were created or analyzed in this study. Data sharing is not applicable to this article.

## Abbreviations

The following abbreviations are used in this manuscript:

APC	Antigen-presenting cell
ApoE	Apolipoprotein E
BBB	Blood–brain barrier
BNCT	Boron neutron capture therapy
BPA	Boronophenylalanine
CD	Cluster of differentiation
CD47	Cluster of differentiation 47
CRISPR	Clustered regularly interspaced short palindromic repeats
DARPins	Designed ankyrin repeat proteins
EGF	Epidermal growth factor
EISA	Enzyme-instructed intracellular self-assembly
EPR	Enhanced permeation and retention
FeOx	Iron oxide
IL-2	Interleukin-2
LET	Linear energy transfer
MHC	Major histocompatibility complex
MMG-1	Mycobacterial monomycoloyl glycerol analogue 1
MRI	Magnetic resonance imaging
NADPH	Nicotinamide adenine dinucleotide phosphate
NK	Natural killer
NP	Nanoparticle
PD-L1	Programmed death-ligand 1
PDPA	Poly(2-diisopropylaminoethyl methacrylate)
PEG	Polyethylene glycol
PEGylation	Polyethylene glycol surface modification
PCL	Poly(ε-caprolactone)
PCL-b-PF	Poly(ε-caprolactone)-b-poly(1-O-acryloyl-β-D-fructopyranose)
PF	Poly(1-O-acryloyl-β-D-fructopyranose)
PGA	Polyglycolic acid
PGAs	Polyglycolic acids
PLA	Polylactic acid
PLAs	Polylactic acids
PLGA	Poly(lactic-co-glycolic acid)
rhGH	Recombinant human growth hormone
siRNA	Small interfering RNA
SOAT2	Sterol O-acyltransferase 2
ssDNA	Single-stranded DNA
ssRNA	Single-stranded RNA
TME	Tumor microenvironment
mPEG-PLA	Methoxy poly(ethylene glycol)-poly(lactic acid)
